# Novel genomic targets of valosin-containing protein in protecting pathological cardiac hypertrophy

**DOI:** 10.1038/s41598-020-75128-z

**Published:** 2020-10-22

**Authors:** Ning Zhou, Xin Chen, Jing Xi, Ben Ma, Christiana Leimena, Shaunrick Stoll, Gangjian Qin, Charles Wang, Hongyu Qiu

**Affiliations:** 1 Division of Physiology, Department of Basic Sciences, School of Medicine, Loma Linda University, Loma Linda, CA 92350 USA; 2 Division of Cardiology, Department of Internal Medicine, Tongji Hospital, Tongji Medical College, Huazhong University of Science and Technology, Wuhan, 430000 China; 3 Center for Genomics and Department of Basic Sciences, School of Medicine, Loma Linda University, 11021 Campus Street, AH 120/104, Loma Linda, CA 92350 USA; 4 Department of Biomedical Engineering, School of Medicine and School of Engineering, University of Alabama At Birmingham, Birmingham, AL 35294 USA; 5 Center of Molecular and Translational Medicine, Institution of Biomedical Science, Georgia State University, Petit Research Center, Room 588, 100 Piedmont Ave, Atlanta, GA 30303 USA

**Keywords:** Biochemistry, Molecular biology, Physiology, Cardiology

## Abstract

Pressure overload-induced cardiac hypertrophy, such as that caused by hypertension, is a key risk factor for heart failure. However, the underlying molecular mechanisms remain largely unknown. We previously reported that the valosin-containing protein (VCP), an ATPase-associated protein newly identified in the heart, acts as a significant mediator of cardiac protection against pressure overload-induced pathological cardiac hypertrophy. Still, the underlying molecular basis for the protection is unclear. This study used a cardiac-specific VCP transgenic mouse model to understand the transcriptomic alterations induced by VCP under the cardiac stress caused by pressure overload. Using RNA sequencing and comprehensive bioinformatic analysis, we found that overexpression of the VCP in the heart was able to normalize the pressure overload-stimulated hypertrophic signals by activating G protein-coupled receptors, particularly, the olfactory receptor family, and inhibiting the transcription factor controlling cell proliferation and differentiation. Moreover, VCP overexpression restored pro-survival signaling through regulating alternative splicing alterations of mitochondrial genes. Together, our study revealed a novel molecular regulation mediated by VCP under pressure overload that may bring new insight into the mechanisms involved in protecting against hypertensive heart failure.

## Introduction

Heart failure is a leading cause of death, despite the availability of effective therapeutic options^1, 2^. Cardiac hypertrophy, in response to pressure overload, such as chronic hypertension, is a significant predictor for heart failure development, and also, an independent risk factor for myocardial infarction, arrhythmia, and sudden death^3, 4^. Despite intensive research efforts over several decades, the molecular mechanisms underlying hypertensive heart failure remain mostly unknown. Therefore, it has become necessary to identify novel targets involved in cardiac hypertrophy's pathogenesis and its transition to heart failure.

Our previous studies found that valosin-containing protein (VCP), an ATPase-associated protein^5, 6^, plays a critical role in cardiac protection against stress^7–9^. Our recent studies further indicate a strong link between the down-regulation of VCP expression and pressure overload-induced cardiac hypertrophy in chronic transverse aortic constriction (TAC) mouse model^10^. Moreover, we found that cardiac-specific overexpression of the VCP in transgenic mice (VCP TG) significantly attenuated the pathological cardiac hypertrophy compared to wild type (WT) mice^10^. These results together strongly suggest a protective role of the VCP against pressure overload-induced cardiac stress. However, despite these exciting functional findings, the regulatory mechanisms involved are still not fully understood.

Using RNA sequencing (RNA-seq), the present study aimed to elucidate the gene regulations conferred by the VCP involved in the protection against the pathogenesis of cardiac hypertrophy induced by TAC. A comprehensive analysis of RNA-seq transcriptome was performed using the heart tissues derived from TAC and sham WT mice to explore the molecular alterations involved in cardiac hypertrophy development. We also used the VCP TG mouse model to identify genes and signaling pathways regulated explicitly by VCP in the heart in response to pressure overload.

## Results

### VCP elicits a distinct stress-associated transcriptomic alteration upon pressure overload in the TG mouse hearts

To determine the transcriptomic regulations underlying pathological cardiac hypertrophy development, both WT and VCP TG adult mice were subjected to TAC for two weeks (2W) to induce pressure overload on the mouse hearts. As shown in our previous publication^10^, compared with sham-operated mice, WT mice developed significant cardiac hypertrophy with a preserved cardiac function after 2W TAC. In contrast, these cardiac hypertrophic alterations were not in VCP TG mice. Since there was no significant difference in cardiac morphology and contractile function between VCP TG and WT mice in the sham groups, these data indicated that VCP mediates a protective mechanism, specifically against pathological cardiac hypertrophy induced by TAC.

A RNA-seq followed by a comprehensive analysis was used to detect the cardiac transcriptomes using the left ventricular (LV) tissues obtained from these TAC and sham mice. We first compared the transcriptomic difference between WT and VCP TG mice, either in the sham or 2W TAC conditions, respectively, with serval independent analyses. As shown in Fig. S1a, b, the principal component analysis (PCA) showed a clear separation of transcriptomic profile between VCP TG and WT mouse groups at both sham and TAC conditions. By using a hierarchical clustering analysis (HCA), we also found a distinct difference in the whole transcriptome between the conditions of sham and 2W TAC when the VCP TG were compared to WT mice (Fig. S1c, d).

**Figure 1:**
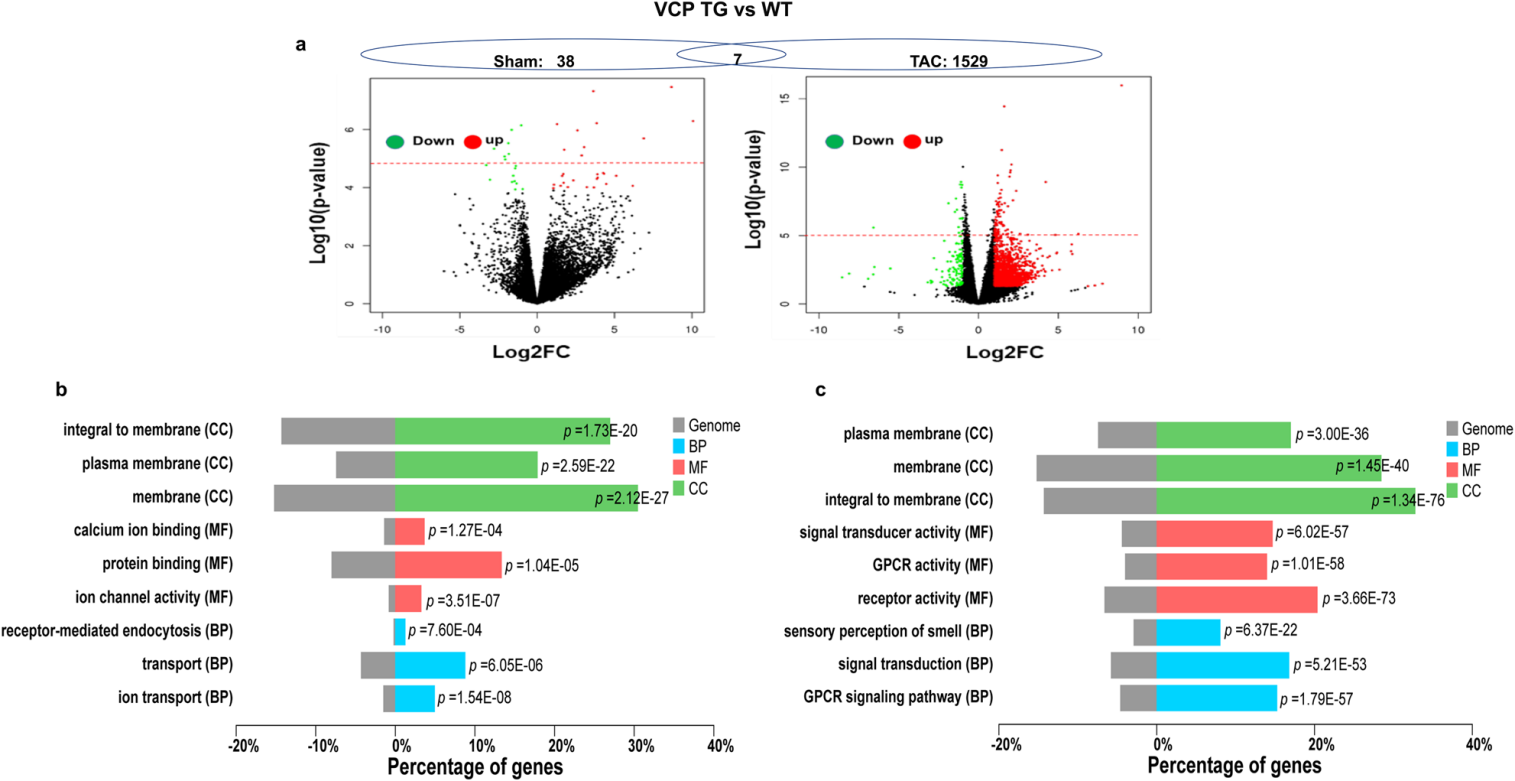
VCP-mediated transcriptomic alterations are different between the sham and 2 weeks (2W) TAC conditions. (**a**) The volcano plot of DEGs between VCP TG and WT mice at the sham and 2W TAC conditions. Red and green dots represent up- and down-regulated in the VCP TG group, respectively. The red dash line represents the threshold of FDR. The dot above the line are genes with FDR < 0.05**.** n = 3–4/group. (**b**), (**c**) GO functional analysis of DEGs induced by VCP between the sham and TAC conditions. GO functions of DEGs based on the FC > Log2 and *p* < 0.05 by comparing VCP TG with WT mice at the condition of sham (**b**) and 2W TAC (**c**), in terms of the cellular components (CC, green), molecular function (MF, red) and biological process (BP, Blue).

To determine the differentially expressed genes (DEGs) between VCP TG and WT mouse hearts, we used two thresholds to analyze the RNA-seq data. By using a fold-change (FC) of more than 2 and a *p* value of less than 0.05 as a threshold, 950 DEGs were identified between VCP TG and WT at sham condition, while 1837 DEGs were identified between these two groups at the end of 2W TAC. To determine the most significant DEGs, we used an even stricter threshold based on the false discovery rate (FDR) < 0.05, which gave rise to 45 DEGs between the VCP TG and WT mice in the sham control, while 1536 DEGs were identified as significant genes between two groups at the end of 2W TAC. These DEGs were displayed in a Volcano plot, which combined the ANOVA test to visualize the statistically significant genes with massive magnitude changes (Fig. 1a). In addition, Gene Ontology (GO) functional analysis was performed to classify the groups of DEGs in terms of their cellular components (CC), molecular function (MF), and biological process (BP) according to the *p* value**.** As shown in Fig. 1b, c, compared to the VCP TG with WT mice, the top enriched CC groups were similar between the sham and TAC conditions as both were predominately involved in membrane proteins. However, the GO analysis based on the MF and BP showed a remarkable difference between the sham and TAC conditions. The VCP induced DEGs at the sham condition were related to the ion binding, transfer, and activity as well as protein binding (Fig. 1b), while the DEGs induced by VCP under the TAC condition were involved the G protein-coupled receptors (GPCRs) and their signaling transductions (Fig. 1c). These data indicate that the gene regulations of the VCP are different between the sham and under the stress conditions.

We further compared the top DEGs between the VCP TG and WT mice detected at both sham and TAC conditions. As shown in Table S1, based on FC, the top DEGs were remarkably different between two states, and the DEGs with the highest FCs were further validated by qRT-PCR (Fig. S2). We also compared the alterations of those most significant DEGs detected in VCP TG mice at the sham condition with their corresponding alterations at the 2W TAC condition. As shown in Table S2, based on FDR < 0.05, among the 45 most significant DEGs induced by VCP at the sham condition, only 7 of them were detected at the TAC groups (highlighted with the bold). These included three genes related to DNA, metal and protein binding (*Hfm1, Cntnap2, Zfp616*), one gene involved in transmembrane protein interaction and receptor transport *(Mamdc4),* and three uncharacterized genes* (Fam11b, BC049715, Fsip2)*. These data together demonstrated that VCP elicits a distinct transcriptomic alteration upon the pressure overload, indicating a stress-specific regulatory role of VCP in the heart. We also examined the corresponding alteration of these seven overlapping DEGs in WT mice under the TAC stress. As shown in Table S2, the modifications of these seven DEGs in WT between 2W TAC vs sham control were opposite to those detected in VCP TG sham mice vs WT sham.

Additionally, to determine the potential association between the VCP-mediated gene regulation and the inhibition of cardiac hypertrophy, we compared the detected DEGs during the development of cardiac hypertrophy secondary to the 2W TAC with those DEGs induced by the VCP overexpression at the sham condition. Based on FDR < 0.05 and FC > 2, 22 overlapping significant DEGs were found between the comparison of WT 2W TAC vs. WT sham and the comparison of VCP TG sham vs WT sham (Table 1). Interestingly, the alterations of these overlapping DEGs in VCP TG mouse hearts showed an opposite manner to those in 2W TAC WT mice when both were compared to WT sham. As shown in Table 1, there were ten DEGs upregulated by 2W TAC in WT mice, but they were downregulated in VCPTG vs WT sham mouse hearts. These DEGs included the genes involved in transmembrane protein interaction and receptor transport (such as *Adam8, Sh3tc1, Mamdc4, Lrrc47, Spns2, Plscr2*); and the genes related to the regulation of fetal development and cell cycle and fate (such as *Sox17, Rgcc, Tacc2, and Fas*). In contrast, 12 DEGs were downregulated in WT after 2W TAC, but they were found to be upregulated in VCP TG mouse heart vs sham. These DEGs included the genes related to the DNA, metal, and protein binding (such as *Erbb4, Hfm1, Dcdc5, Sytl2, Cntnap2, Zfp616*), the oxygen transport (*Hbb-bt)*, and other genes related to cell development and motility, and organization of microtubules (such as *Upk1b, Fsip2, Ccdc40, and Spag17*). *ErbB4*, one of the tyrosine kinase receptors of the growth factor neuregulin-1 (NRG1), was also downregulated in 2W TAC while was upregulated in the VCP TG sham mice.

**Table1:** The overlapped DEGs between 2W TAC WT and VCP TG compared to WT sham.

Ensembl ID	Gene symbol	WT 2W TAC vs WT sham		VCPTG sham vs WT sham		Gene names
		log2FC	FDR	log2FC	FDR	
ENSMUSG00000025473	*Adam8*	3.026	0.00004	− 2.789	0.00739	*A disintegrin and metalloproteinase domain 8*
ENSMUSG00000025902	*Sox17*	2.336	0.00004	− 2.108	0.01023	*SRY-box transcription factor 17*
ENSMUSG00000022018	*Rgcc*	1.739	0.00147	− 1.864	0.00571	*Regulator of cell cycle*
ENSMUSG00000036553	*Sh3tc1*	1.665	0.00013	− 1.463	0.03648	*SH3 domain and tetratricopeptide repeat-containing protein 1*
ENSMUSG00000026941	*Mamdc4*	1.577	0.00015	− 1.650	0.00260	*MAM domain containing 4*
ENSMUSG00000040447	*Spns2*	1.474	0.00041	− 1.425	0.02098	*Sphingolipid transporter 2*
ENSMUSG00000030852	*Tacc2*	1.383	0.00225	− 1.417	0.04971	*Transforming acidic coiled-coil containing protein 2*
ENSMUSG00000029028	*Lrrc47*	1.305	0.00000	− 1.036	0.00231	*Leucine rich repeat containing 47*
ENSMUSG00000032372	*Plscr2*	1.213	0.00753	− 1.334	0.03989	*Phospholipid scramblase 2*
ENSMUSG00000024778	*Fas*	1.200	0.03149	− 1.835	0.00956	*Cell surface death receptor*
ENSMUSG00000062209	*Erbb4*	− 1.216	0.00348	1.614	0.02830	*Erb-B2 receptor tyrosine kinase 4*
ENSMUSG00000073940	*Hbb-bt*	− 1.355	0.03057	3.666	0.04569	*Hemoglobin, beta adult t chain*
ENSMUSG00000043410	*Hfm1*	− 2.543	0.02624	3.215	0.04568	*Helicase for meiosis 1*
ENSMUSG00000074981	*Dcdc5*	− 2.559	0.00000	1.746	0.00740	*Double cortin domain containing 5*
ENSMUSG00000030616	*Sytl2*	− 2.885	0.00166	3.031	0.00712	*Synaptotagmin like 2*
ENSMUSG00000075249	*Fsip2*	− 2.937	0.00005	2.874	0.01015	*Fibrous sheath interacting protein 2*
ENSMUSG00000039419	*Cntnap2*	− 2.954	0.00642	3.904	0.03127	*Contactin associated protein 2*
ENSMUSG00000069476	*Zfp616*	− 3.345	0.00135	4.265	0.02804	*Zinc finger protein 616*
ENSMUSG00000039963	*Ccdc40*	− 3.652	0.00209	3.843	0.00231	*Coiled-coil domain containing 40*
ENSMUSG00000095996	*Gm16513*	− 3.986	0.00952	4.469	0.04030	*Gm16513*
ENSMUSG00000027867	*Spag17*	− 4.449	0.00000	3.887	0.02804	*Sperm associated antigen 17*
ENSMUSG00000049436	*Upk1b*	− 6.224	0.00024	4.315	0.02804	*Uroplakin 1B*

### VCP induces specific DEGs involving GPCRs in response to the pressure overload in the mouse hearts

Considering the regulation of VCP is stress-associated, we further explored the specific gene regulation in response to pressure overload by examining the DEGs between the 2W TAC mice and their corresponding sham controls in either VCP TG mice or WT mice. The comparison was performed to explore the different responses to TAC between two groups.

**Figure 2:**
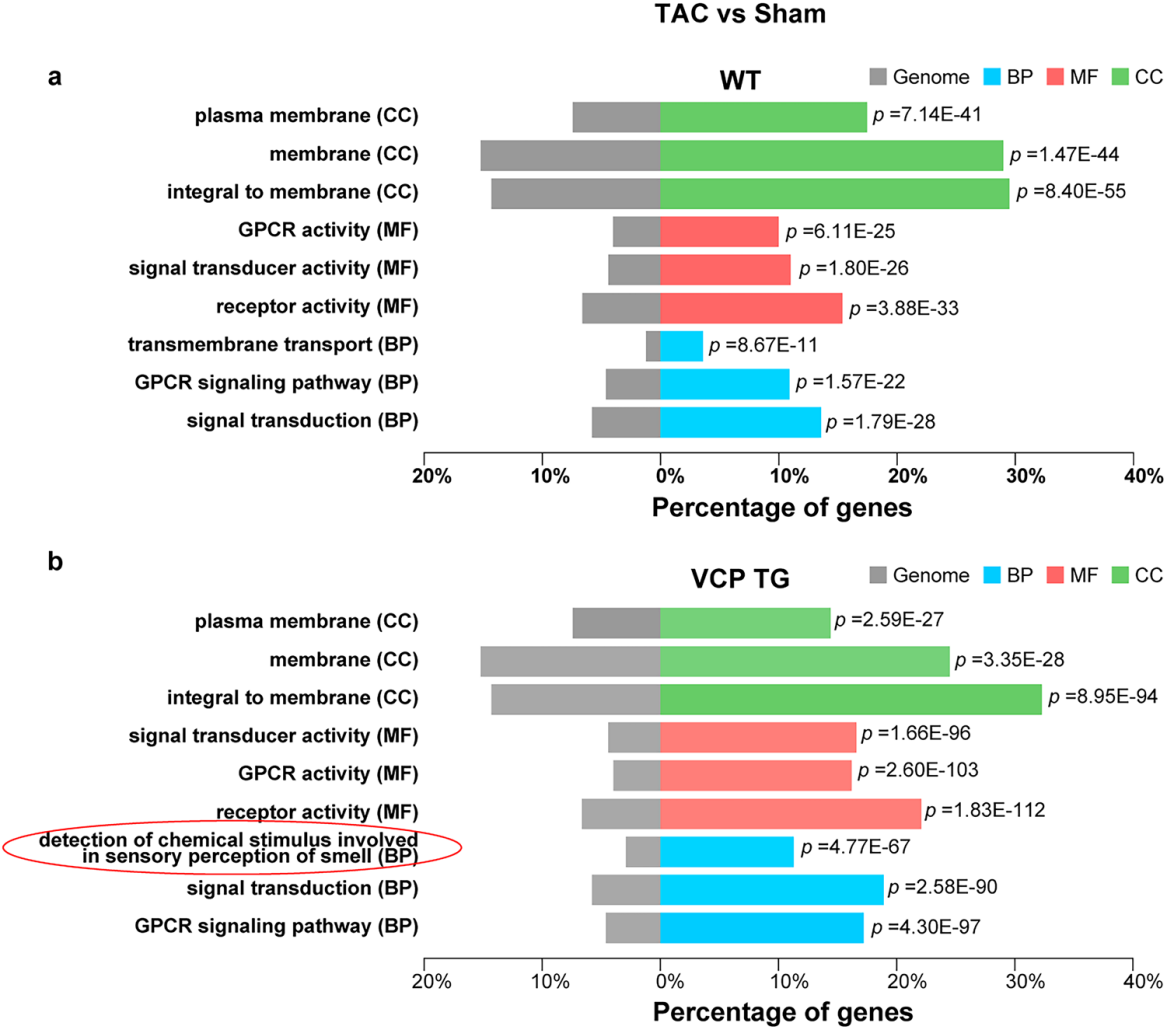
GO functions of DEGs in response to 2W TAC were different between VCP TG and WT mice. GO analysis of DEGs based on the comparison of 2 weeks (2W) TAC vs sham in both WT (**a**) and VCP TG mice (**b**), respectively. The circled group indicated a distinct GO “component” in sensory perception of smell in the VCPTG mice in response to 2W TAC.

As shown in Fig. 2a, b, the majority of GO "function" of DEGs were similar between the VCP TG and WT mice within the comparison of the 2W TAC with sham. However, there was a distinct GO "component" in sensory perception of smell in the VCP TG mice in response to 2W TAC (Fig. 2b). In addition, as shown in Table 2, based on FDR < 0.05, the top 20 DEGs were upregulated genes in 2W TAC VCP TG mice compared with their sham controls, while most of the top DEGs were downregulated genes in 2W TAC vs sham in the WT. Consistent with the results of the GO analysis, among these top 20 ranked DEGs in VCP TG mice, more than half (13 out of 20) of the genes belonged to the olfactory receptor family (*Olfr787, Olfr193, Olfr1311, Olfr1299, Olfr1303, Olfr998, Olfr1231, Olfr1448, Olfr73, Olfr507, Olfr782, Olfr498, Olfr1023*), plus two vomeronasal one receptors (*V1rd19 and Vmn1r183*) (Table 2).

**Table 2:** Top differentially expressed genes between 2-week TAC and sham controls.

Type of mouse	Gene	log2FC	*p* value	FDR
WT	*Upk1b*	− 6.2244	0.0000	0.0002
	*Gpr39*	− 5.8972	0.0004	0.0097
	*Neurod4*	− 5.8022	0.0000	0.0005
	*Folh1*	− 5.7298	0.0000	0.0002
	*Vmn2r82*	− 5.7233	0.0000	0.0014
	*Tex16*	− 5.5070	0.0000	0.0002
	*Fam110c*	− 5.4276	0.0001	0.0044
	*Olfr1079*	− 5.4015	0.0001	0.0043
	*Tat*	− 5.2577	0.0001	0.0042
	*Odam*	− 5.2269	0.0009	0.0157
	*Vmn2r96*	− 5.1002	0.0001	0.0045
	*Cysltr2*	− 5.0477	0.0004	0.0091
	*Zfp936*	− 5.0357	0.0005	0.0109
	*Vmn2r81*	− 5.0169	0.0012	0.0195
	*Vmn1r2*	− 5.0115	0.0037	0.0408
	*Opn5*	− 4.9529	0.0002	0.0063
	*Olfr1373*	4.9260	0.0028	0.0333
	*Olfr181*	− 4.9212	0.0050	0.0487
	*Pcdha7*	− 4.8860	0.0012	0.0188
	*Mup20*	− 4.8626	0.0024	0.0304
VCP TG	*Smok3a*	6.2940	0.0000	0.0024
	*Olfr787*	6.1800	0.0000	0.0053
	*Olfr193*	6.0027	0.0001	0.0160
	*Olfr1311*	5.8636	0.0001	0.0176
	*Olfr1299*	5.8278	0.0003	0.0312
	*V1rd19*	5.7917	0.0005	0.0378
	*Olfr1303*	5.7071	0.0005	0.0368
	*Olfr998*	5.6843	0.0004	0.0327
	*Ms4a13*	5.6784	0.0003	0.0284
	*Gjb6*	5.6109	0.0003	0.0304
	*Obp1b*	5.5921	0.0008	0.0483
	*Olfr1231*	5.5878	0.0007	0.0448
	*Olfr1448*	5.5739	0.0005	0.0368
	*Olfr73*	5.5693	0.0004	0.0355
	*Olfr507*	5.5203	0.0007	0.0448
	*Vmn1r183*	5.4645	0.0007	0.0469
	*Olfr782*	5.3760	0.0002	0.0259
	*Olfr498*	5.3532	0.0001	0.0164
	*Olfr1023*	5.3295	0.0004	0.0347
	*Ugt1a10*	5.2489	0.0001	0.0165

*Upk1b*: Uroplakin 1B.

*Gpr39*: G protein-coupled receptor 39.

*Neurod4*: Neuronal differentiation 4.

*Folh1*: Folate hydrolase 1.

*Vmn2r*: Vomeronasal 2, receptor.

*Tex16*: Testis expressed gene 16.

*Fam110c*: Family With sequence similarity 110 member C.

*Olfr*: Olfactory receptor.

*Tat*: Tyrosine aminotransferase.

*Odam*: Odontogenic, ameloblast associated.

*Cysltr2*: Cysteinyl leukotriene receptor 2.

*Zfp936*: Zinc finger protein.

*Vmn1r*: Vomeronasal 1, receptor.

*Opn5*: Opsin 5.

*Pcdha7*: Protocadherin alpha 7.

*Mup20*: Major urinary protein 20.

*Smok3a*: Sperm motility kinase 3A.

*V1rd19*: Vomeronasal 1 receptor, D19.

*Ms4a13*: Membrane spanning 4-domains A13.

*Gjb6*: Gap junction protein beta 6.

*Obp1b*: Odorant-binding protein 1b.

*Ugt1a10*: UDP glucuronosyltransferase family 1 member A10.

WT: Wild-type mice.

VCP TG: VCP transgenic mice.

FC: Fold-changes.

FDR: The false discovery rate.

**Figure 3:**
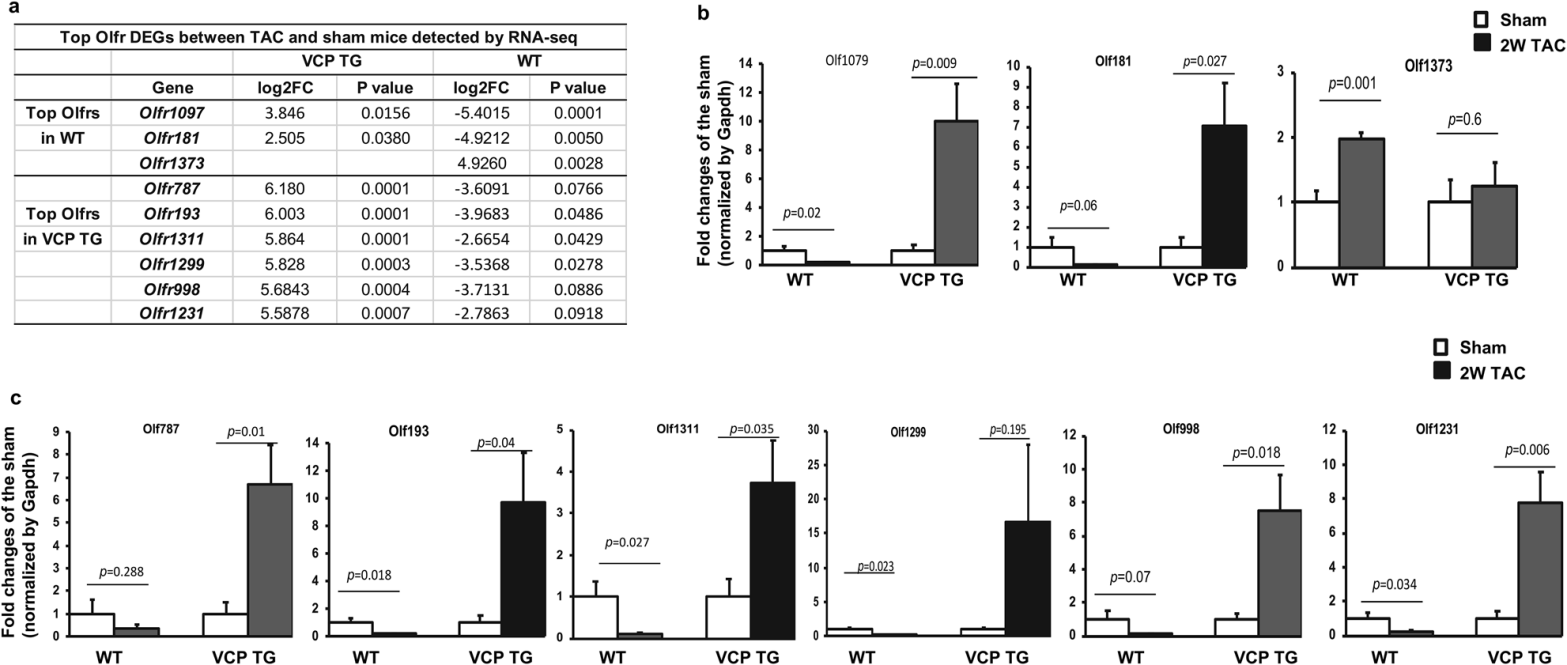
The top Olfr DEGs detected by RNA-seq were validated by qRT-PCR. (**a**) The top Olfr DEGs between 2W TAC vs sham detected by RNA-seq in WT and VCP TG mice respectively. (**b)** qRT-PCR shows the alterations of the top Olfr DEGS between 2W TAC vs sham mice detected in WT mice and their corresponding changes in VCP TG mice. (**c**) qRT-PCR shows the alterations of the top Olfr DEGS between 2W TAC vs sham mice detected in VCP TG mice and their corresponding changes in WT mice. n = 4–5/group. Gapdh was used as the control to normalize the targeted genes.

Interestingly, we found that a few top Olfr DEGs presented in Table 2 were overlapped between VCP TG and WT mice, but showing a different or an opposite change, as using FC more than 2 and p-value less than 0.05 as a threshold. As shown in Fig. 3a, when comparing the 2W TAC with their sham controls, *Olfr1097* and *Olfr181* were found to be the top significant Olfr DEGs that were downregulated in WT mice, but they were upregulated in VCP TG mice (log2FC − 5.4 in WT vs log2FC 3.8 in VCP for *Olfr1097*; and log2FC − 4.9 in WT vs log2FC 2.5 in VCP TG for *Olfr181,*respectively). In addition, *Olfr1373* was upregulated in WT, but was not detected in VCP TG mice (Fig. 3a). In contrast, there were several top Olfr DEGs showing significant upregulation in VCP TG mice, but were downregulated in the WT mice, including *Olfr193* (log2FC 6.0 in VCP TG vs log2FC − 3.97 in WT), *Olfr1311* (log2FC 5.86 in VCP TG vs log2FC − 2.66 in WT) and *Olfr1299* (log2FC 5.83 in VCP TG vs log2FC − 3.53 in WT). The different or opposite regulations between VCP TG and WT mice on these top Olfr DEGs identified by RNA-seq were further validated by qRT-PCR (Fig. 3b, c). In addition, we also noticed a few top Olfr genes showing a significant upregulation in VCP TG mice were downregulated in WT mice, but did not reach statistical significance, such as *Olfr787, Olfr1303, Olfr998, Olfr1231, Olfr1448, Olfr73, Olfr507, Olfr782, Olfr498, Olfr1023* (Table 2, Fig. 3c).

### Ingenuity pathway analysis (IPA) IPA predicts VCP to inhibit TAC-induced hypertrophic upstream transcription factor

**Figure 4:**
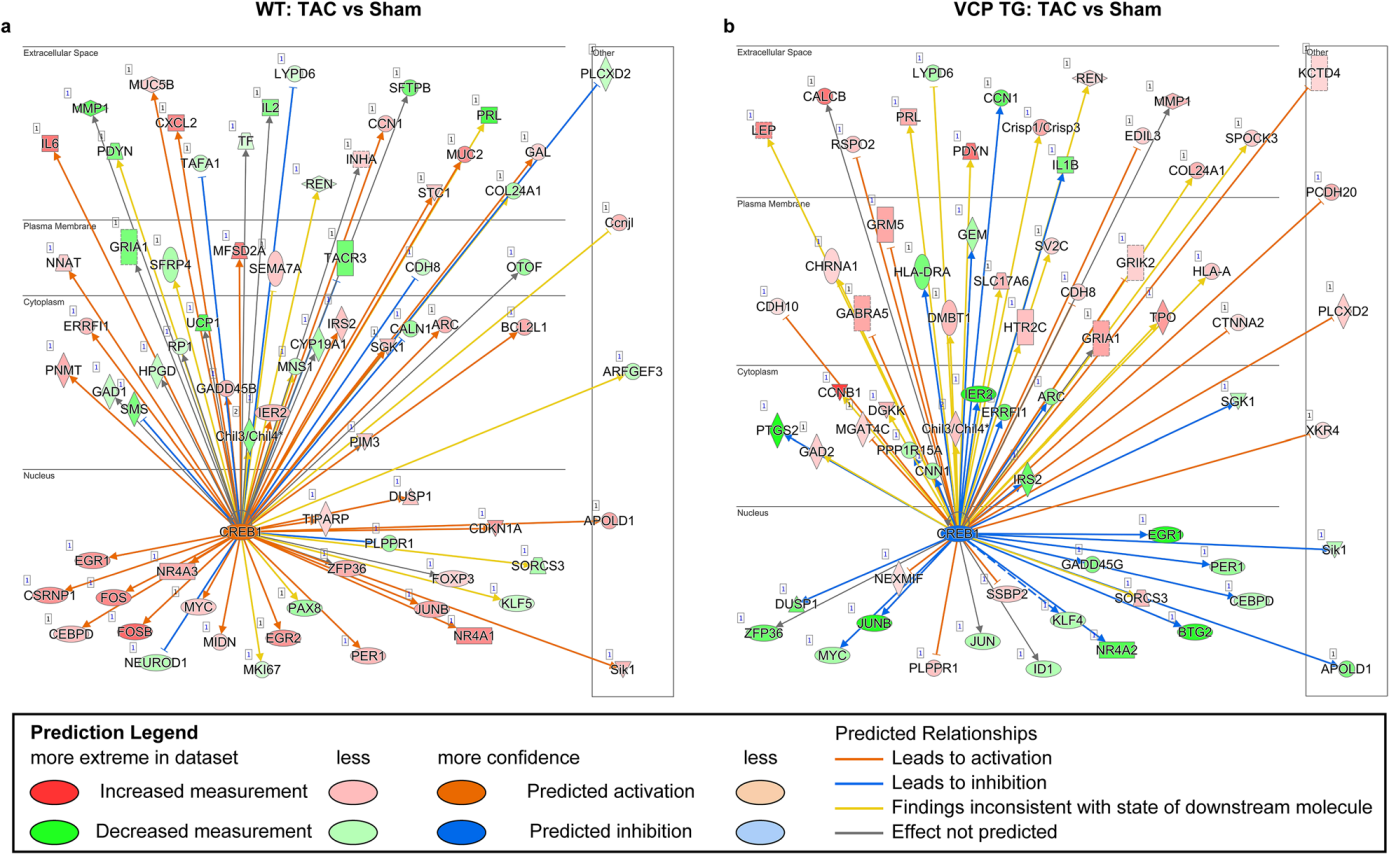
Transcription factor *CREB1* exhibits an opposite regulation in the downstream genes between WT and VCP TG mice in response to 2W TAC. Based on the comparison between 2W TAC and sham controls, in WT and VCP TG mice, respectively, an Ingenuity Pathway Analysis (IPA) (Qiagen’s ) of the RNA-seq data identified *CREB1* as a top transcription factor in both WT and VCP TG mice but showing an opposite regulation between two groups (Data were analyzed through the use of IPA (QIAGEN Inc., https://www.qiagenbioinformatics.com/products/ingenuitypathway-analysis)). *CREB1* mediated signaling was predicted to be activated in WT (**a**) but inhibited in VCP TG mice (**b**).

To further identify the upstream regulators of the DEGs in VCP TG mice, we conducted an IPA to determine the top transcription factors that were associated with the most of significant DEGs detected in both VCP TG and WT groups by using *p*-value was less than 0.05 and FC was greater than 2 as the threshold. Our IPA analysis identified cAMP-responsive element-binding protein 1 (*CREB1*), a phosphorylation-dependent transcription factor, was found to be one of the top transcription factors associated with the significant DEGs in both VCP TG and WT groups, when the TAC mice were compared to their corresponding sham mice. Notably, this transcription factor appeared an opposite regulation on the downstream genes between VCP TG and WT mice. As shown in Fig. 4a, b, compared to the sham controls, *CREB1* was predicted to be activated in the WT mice under the treatment of 2W TAC (Fig. 4a), but to be inhibited in 2W TAC VCP TG mice (Fig. 4b).

### VCP is predicted to modulate the alternative splicing of mitochondrial proteins under the TAC stress

To determine whether VCP regulated differential transcript splicing in response to the cardiac stress, we conducted an alternative splicing analysis based on the differentially expressed transcripts (DETXs) between VCP TG and WT mice at both sham and 2W TAC conditions by using a recently developed count-based statistical model, LeafCutter^11^. The alternatively excised intron clusters were identified by the LeafCutter model and intron usage as counts or proportions were summarized. With this analysis, 18 DETXs were predicted in the sham groups, and 39 DETXs were predicted in TAC groups between VCP TG and WT mice (Table S3). Among these DETXs**,** two DETXs, e.g., *Sorbs1 (Sorbin and SH3 domain-containing protein 1*), a gene regulating cell adhesion and cytoskeletal formation, and *Ttn*, a cellular structure gene, were detected in VCP TG vs WT at both the sham and 2W TAC conditions (Table S3). These two DETXs were also found in the WT 2W TAC mice when they were compared to WT sham controls (Table S3).

**Figure 5:**
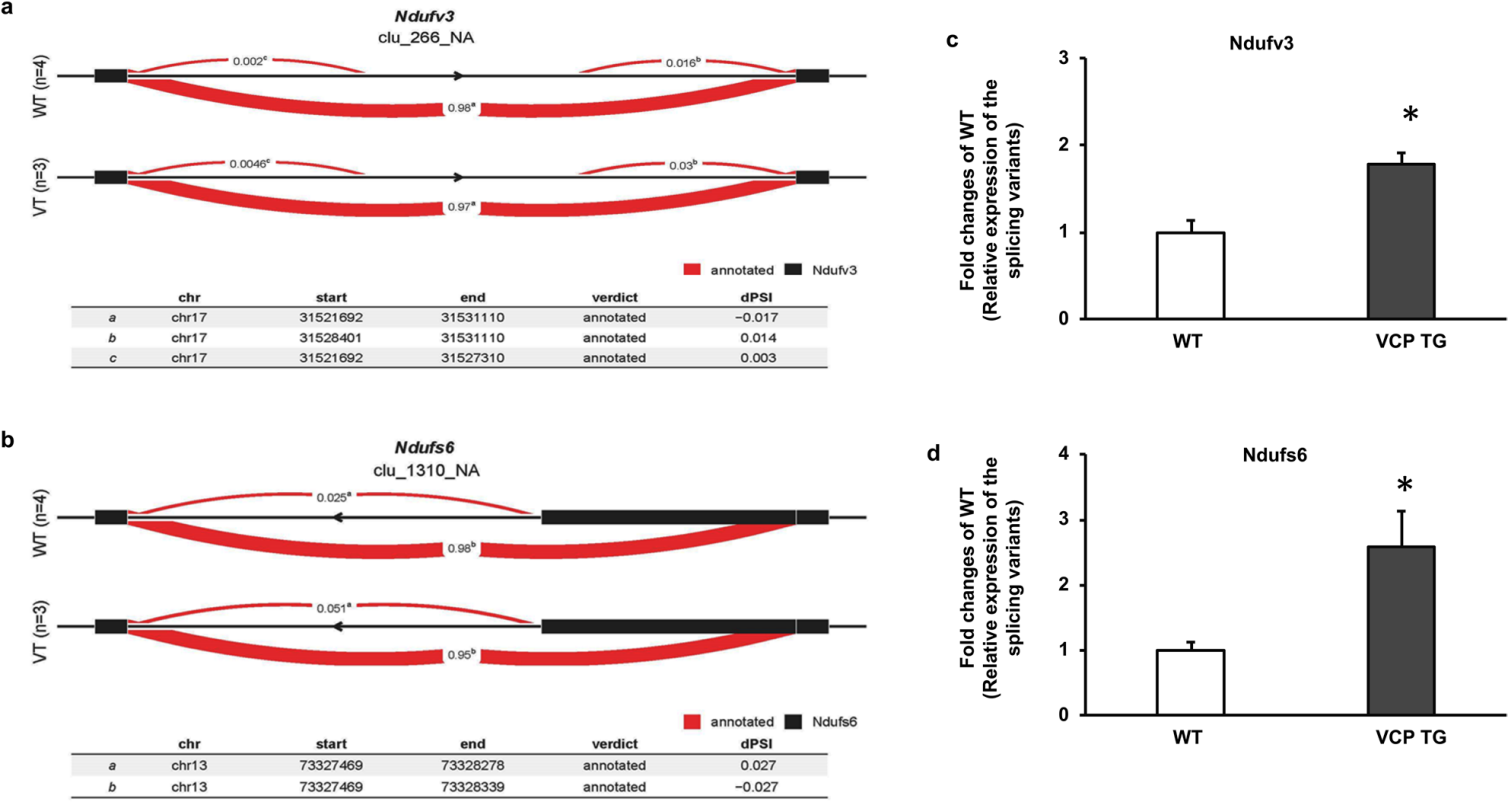
VCP involves in the alternative splicing of mitochondrial proteins under the stress. (**a**), (**b**) Representative LeafCutter cluster plots of the alternative splicing generated by LeafViz for the *Ndufv3* (**a**) and *Ndufs6* (**b**) in VCP TG vs WT at 2W TAC based on FDR < 0.05. Differential splicing was measured by the change in percent spliced in (dPSI). These two DETXs showed differential usage of a mutually exclusive exon between VCP TG and WT groups. (**c**), (**d**) Representative qRT-PCR results show the relative expression of splice isoforms by using two pairs of primers *: *p* < 0.05, n = 4/group.

In addition, among the DETXs between VCP TG and WT under 2W TAC conditions, two DETXs belonging to the subunits of NADH:ubiquinone oxidoreductase (complex I), e.g., *NADH dehydrogenase ubiquinone flavoprotein 3* (*Ndufv3*) and *NADH dehydrogenase iron-sulfur protein 6* (*Ndufs6*) were detected (Table S3). We further used LeafViz to visualize the significant splicing events for these two DETXs in each group^11^. The splicing events of *DETXs* detected in WT and VCP TG at 2W TAC were identified based on differential usage of a mutually exclusive exon. Differential splicing was measured by a change in the percentage of spliced in dPSI using FDR < 0.05. In Fig. 5a, b, the splicing events of *Ndufv3* and *Ndufs6* displayed different profiles in the alternative intron-excision options in two groups by different dPSI (Fig. 5a, b).

qRT-PCR was also used to validate the alterations of alternative splicing for each gene by measuring the relative expression level of the corresponding splicing variants using two primer sets^12^. In Fig. 5c, d, the qPCR results showed different ratios of expression for the splicing variants between WT and VCP TG mice at 2W TAC, which supported the prediction of LeafCutter analyses (Fig. 5a, b).

## Discussion

Our previous results indicated VCP was a promising therapeutic candidate. It has been demonstrated in the TG mice that an increase of VCP prevents the stress-induced pathological cardiac deterioration with fewer side effects on normal unstressed hearts^10^. The current study further investigated the molecular mechanisms by which VCP protects the heart against pressure overload-induced cardiac hypertrophy. Our results from this study revealed a comprehensive transcriptomic characterization of the VCP under cardiac stress, which brought new insight into cardiac protection's molecular mechanisms.

Our results from the GO analysis revealed VCP-induced DEGs at the sham condition were related to DNA, ion and protein binding, and protein transfer and activity. We found a few DEGs related to DNA, metal, and protein binding was upregulated in VCP TG vs WT at both sham and 2WTAC conditions, included *Hfm1, Cntnap2, Zfp616*. Simultaneously, *Mamdc4,* a gene involved in receptor transport, was downregulated in VCP TG at sham condition, but increased under TAC condition. It was notable that these DEGs showed an opposite alteration between the 2W TAC WT and VCP TG sham as they were compared to the WT sham mice. These results shed some light in understanding the protective effect of the VCP against pressure-overload-induced hypertrophy; however, the role of these genes remains largely unknown.

Our results also showed that overexpression of the VCP could induce a gene regulation that would resist the molecular alterations induced by 2W TAC in WT. For example, while 2W TAC in WT mice induced an upregulation on the genes involved in transmembrane protein interaction and receptor transport as well as the regulation of fetal development and cell cycle /fate (*Adam8, Sh3tc1, Mamdc4, Lrrc47, Spns2, Plscr2, Sox17, Rgcc, Tacc2, and Fas*), these genes were downregulated by the overexpression of VCP. In contrast, an overexpression of the VCP could upregulate the genes related to DNA and protein binding and cell motility (*Erbb4, Hfm1, Dcdc5, Sytl2, Cntnap2, Zfp616*), while 2W TAC downregulated these genes in WT mice. These data indicate that the overexpression of the VCP may play a protective role by preemptively regulating the relative gene expression induced by TAC, thus preventing the TAC-induced pathological signaling. Although these genes' exact role and regulatory mechanism remain largely unknown, evidence has indicated some potential effects of these genes on heart. For example, studies have shown that the downregulation of Erb4 was associated with chronic cardiac hypertrophy secondary to aortic stenosis, which played a role in the transition from compensatory hypertrophy to failure^13^. It was also found that an elevated expression of ADAM8 was associated with vascular diseases in mice and humans^14^ and it acts as a potential surrogate of inflammation, which has been associated with myocardial infarction^15^. These studies support our findings and stimulate future investigation to exploring the molecular mechanism underlying the cardiac protection conferred by VCP.

Notably, the analysis of the transcriptomic profiles showed that VCP-induced gene regulation under stress in 2W TAC mice was dramatically different from those observed in the sham groups when VCP TG mice were compared to WT mice. These results indicate that VCP acts as a stress-associated protein and plays a protective role, specifically in the stressed hearts that are distinct from the normal unstressed hearts. These data support our previous phonotypic findings of an overexpression of VCP protected the heart against pressure overload-induced pathological hypertrophy, but it did not affect cardiac growth in normal hearts^10^.

Given the strong link between the pressure overload-induced cardiac hypertrophy and heart failure, we focused on the cardiac transcriptomic characterization of VCP in response to 2W TAC. One of our novel findings was that, among the top regulated DEGs, a group of olfactory receptors (ORs) and vomeronasal receptors (VRs) were found to be significantly upregulated in the VCP TG mice at 2W TAC when compared to their shams. Interestingly, some of these ORs were downregulated in WT in response to the 2W TAC. The opposite regulation on these genes between the VCP TG and WT mice indicates that these receptors may play an essential role in the VCP-mediated cardiac protection against the pressure-overload stress. We also noticed that some DEGs were only specifically regulated in WT mice, indicating that multiple signaling pathways may be involved in the pressure-overload induced alterations of the transcripts in WT. Although ORs and VRs have been reported to be located in the heart muscle cells^16, 17^, as known odor receptors in the neurons, these genes' roles in the heart has not been recognized. It has been shown in other tissues that ORs were involved in the activation of the olfactory-type G protein, which in turn activated the lyase-adenylate cyclase that converts ATP into cyclic AMP (cAMP)^18^, participating in the transfer of calcium and sodium ions into the cell^19^. Other ORs located in the immune system has been linked to the death of some types of leukemia cells^20^. Although both ORs and VRs are GPCRs, these receptors are distantly related to the primary olfactory system's receptors, highlighting their different roles^18, 21^. Our results showed for the first time the link between VCP and these ORs and VRs, which suggests a potential protective role of these genes in preventing pressure overload-induced cardiac hypertrophy. These data open a new research direction to explore the role of these receptors in cardiac protection.

Although our previous study has shown a significant increase of Nppa and Nppb in the WT TAC vs sham by qPCR, which was accompanied by multiple corresponding physiological and histological alterations, validated the TAC model^10^, this significant increase was not detected by RNA-seq. Despite the multiple potential reasons, one of the failed detections may be due to the very low expression level of these fetal genes in the normal adult ventricles, which may not reach the expression level to be detected reliable by RNA-seq at our sequencing depth. We also would like note that, for some scarcely expressed genes, it is known that sometimes there is an inconsistency between the RNA-seq and qPCR results, since qPCR is more sensitive. In addition, our qPCR results showed that Nppa is relatively increased in VCP TG vs WT sham mice. Although Nppa was detected to be dramatically increased in the pathological cardiac hypertrophy as reported from our and others’ previous studies, an increased ventricular expression of Nppa was not necessarily correlated with cardiac hypertrophy, particularly with small amount of increase. It has been shown that hypertrophy can occur in the absence of increased ventricular Nppa, and increased levels of Nppa can also occur in the absence of detectable cardiac hypertrophy^22^, indicating that the cardiac hypertrophy is the result of a multifactorial process. On the other hand, transgenic overexpression of Nppa tended to protect against hypertrophic stimuli^23^. Since the regulatory mechanisms underlying the Nppa expression in the adult ventricles and the subsequent reactivation in the diseased heart in vivo have not been resolved satisfactorily, the role of VCP in these processes need further investigation.

Another novel finding from this study was identifying the upstream transcription factors associated with VCP-mediated protection. The IPA analysis identified *CREB1 as* one of the top transcriptional factors that were regulated in both the VCP TG mice and WT mice in response to the 2W TAC, but oppositely. As *CREB1* was predicted to be activated in the WT mice, it was predicted to be inhibited in the VCP TG mice. It is known that *CREB1* is a member of the leucine zipper family of DNA binding proteins and it can be phosphorylated by several protein kinases to induce the transcription of genes in response to hormonal stimulation of the cAMP pathway^24^. Considerable evidence indicates that *CREB1* is involved in cardiac hypertrophy upon stimulation^25, 26^. Our data supported a strong association between the activation of CREB1 signaling and the pressure overload-induced cardiac hypertrophy in the WT mice.

Several studies indicated a direct link between the activation of CREB and GPRCs, which involves in a highly conserved cAMP/PKA/CREB pathway. It has been shown that GPCRs binding with their ligands would lead to the dissociation of the heterotrimeric G protein complex, which subsequently activates or inhibits the transmembrane adenylyl cyclase molecules. The activated cyclase increases cAMP synthesis, which binds to the regulatory subunit of protein kinase A (PKA-R), leading to a dissociation of the catalytic subunits (PKA-C) from tetramers consisting of regulatory and catalytic subunits. Free PKA-C then phosphorylates the substrate proteins, including transcription factor CREB. Phosphorylation of CREB is required for interaction with the CREB-binding protein (CBP) co-activator. The activation of transcription of the genes contain cAMP response element (CRE) sites in their promoters^27–29^. Olfactory receptors (ORs) belong to the GPCRs family and they have been shown to be involved in the regulation of CREB-mediated gene expression^30^. Our data showed that VCP-mediated regulation is highly associated with both, the ORs genes and CREB-mediated signaling, implying a potential link between ORs and CREB activity; however, the precise mechanisms underlaid will need some further studies.

Our previous study has shown that VCP attenuates pathological cardiac hypertrophy by selective inhibiting pressure-overload induced mTORC1/AKT/pS6 signaling^10^. However, the exact molecular mechanisms underlying this regulation remained mostly unknown. The results from this study brought some new insights into the understanding of VCP's regulation on this signaling. First, the most recent studies showed that GPCR signaling inhibited mTORC1^31^. Our RNA-seq data indicated that VCP upregulated a group of GPCR, particularly ORs, which suggested that the inhibitory effect of VCP on mTORC1 under stress could be mediated by the activation of these ORs, thus, providing a potential explanation for our previous findings. Secondly, several studies have indicated a connection between Akt phosphorylation at 308 and the activation of CREB in other tissues, such as neurons and 293 T cells^32, 33^. These studies showed that Akt phosphorylation at 308 (pAkt-308) interacted and co-located with CREB, required for the CREB phosphorylation. These results imply an association between VCP-mediated downregulation of pAKT-308 detected in our previous study and the inhibitive effect of CREB-mediated genes identified in the current study. This further indicates that VCP acts as a new inhibitor for *CREB1* signaling by which the VCP prevents the pressure overload-induced activation of this signaling.

Finally, our results also revealed a novel role of VCP in regulating RNA splicing alterations under the stress of pressure overload. The excision of introns from pre-mRNA is an essential step in mRNA processing. We used a newly developed statistical model, LeafCutter^11^, to detect the potential alternative splicing that may be linked to VCP. This analysis showed that it accurately identified the robust variation in intron excision across conditions with a count-based statistical modeling^11^. Our results showed that two important DETXs associated with the first enzyme complex (Complex I) in the electron transport chain of mitochondria, e.g., *Ndufs6* and *Ndufv3*, were found to be altered in the VCP TG vs. WT mice at 2W TAC. These data strongly supported our previous findings that VCP increased the complex I dependent mitochondrial respiration in the heart^9^. Particularly, DETXs of Ndufv3 and Ndufs6 were only detected in the VCP TAC mouse hearts, but not in WT TAC hearts, indicating a potential specific effect of VCP response to the 2W TAC. We are particularly interested in Ndufv3 and Ndufs6 for the following reasons: first, our previous studies showed that VCP acted as a stress-associated protein that played a protective role in the stressed hearts that are distinct from the normal unstressed hearts. Our current analysis of transcriptomic profiles also showed that VCP-induced gene regulation under stress in the 2W TAC mice was dramatically different from those observed in the sham control mice when VCP TG mice were compared to WT mice. These two DETXs were detected in the 2W TAC in VCP TG, but not in the sham groups when compared to WT. Second, these two DETXs are associated with the complex I of mitochondria, which strongly supported our previous finding that the VCP increased the complex I dependent mitochondrial respiration in the heart. Third, much less alternative splicing sites were detected in these two genes, making it relatively easier for us in the future with more functional validations to identify the possible alternative splicing of the isoforms that are specifically responsive to VCP.

In addition, we found that DETXs that were involved in cell metabolism and energy generation were regulated by VCP, such as, *Sorbs1,* a significant regulator of insulin-stimulated signaling and regulation of glucose uptake^34^. As it has been reported that Sorbs1 is involved in a second signaling pathway required for insulin-stimulated glucose transport^34^, our results may imply a potential link between VCP and cardiac energy metabolism. However, considering that Sorbs1 has multiple start sites and alternately spliced isoforms, which dramatically increase the complexity of regulation and the difficulty in identifying the role of each specific isoform, further investigations are needed to determine the particular regulating effects conferred by VCP. Furthermore, we found that VCP was also involved in regulating the splicing alteration of genes participating in the structure and contractility of the heart muscle, such as *Ttn*. Interestingly, mutations in this gene are associated with familial hypertrophic cardiomyopathy^35^.

As a powerful approach for studying variation in alternative splicing, this analysis allows the identification and quantification of both known and novel alternative splicing events, which may bring new insights into the regulation of VCP. However, it should be noted that the results from such tests are only predictive, and any identified specific splicing variants should be subject to further validation by functional studies. It is particularly applicable for those genes with multiple start sites with many alternate spliced isoforms, such as Sorbs1 and Ttn, for which there may be difficulty in identifying the alteration and the role of each specific isoform.

**Figure 6:**
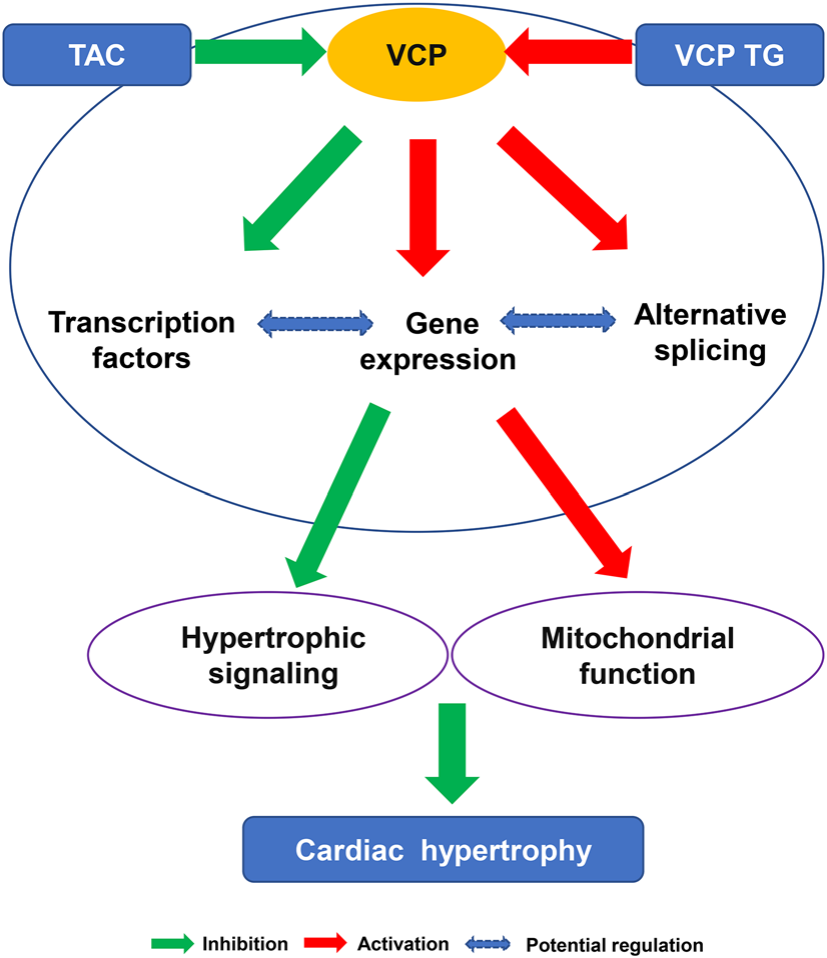
Summary of the gene network regulated by VCP upon pressure overload. The regulatory mechanism underlying the cardiac protection of VCP involved the potential effects at multiple levels including the upstream transcription factors, gene expression and the alternative splicing, which constitute an integrative gene network, inhibiting the hypertrophy signaling and promoting mitochondrial function in the stressed hearts.

In summary, as showed in Fig. 6, our data revealed potential new transcriptomic networks underlying the cardiac protection conferred by VCP, which involved the regulations at multiple levels including the specific hypertrophic transcription factor and genes and the alternative splicing of mitochondrial genes, inhibiting the hypertrophy signaling and promoting mitochondrial function in the stressed hearts.

## Methods

### Study design

To explore the molecular basis of the cardiac protection conferred by the VCP, a cardiac-specific VCP TG mouse model was generated and compared to its litter-mated WT mice. To mimic the pressure overload-induced cardiac stress, both WT and VCP TG mice were subjected to a TAC surgery for 2 weeks as described previously^10^ and a group of mice with the sham operation was the control. Physiological and histological studies confirmed the success of the models. The LV tissues collected from these mice and mRNAs were extracted and used for RNA sequencing. The quality assessment was performed based on external RNA spike-in controls**.** Comparisons were first performed between VCP TG and WT mice at two treatment conditions: sham and 2W post-TAC to determine the different regulation of the cardiac transcriptomics by VCP between the stressed and unstressed conditions, and then performed between sham and TAC in WT and VCP TG mice, respectively, to characterize the genomic regulations involved in cardiac hypertrophy induced in WT mice and the protective regulations in VCP TG mice against the pressure overload-induced cardiac stress. The detailed methods were described as follows.

### Animals and heart tissue samples

A cardiac-specific VCP TG mouse model was generated as described previously^10^, in which the cardiomyocytes showed an increase of VCP by 3.5 folds compared with WT mice^10^. There are no significant differences between the VCP TG and WT mice at the baseline condition at three to six months old. No difference was found between male and female mice in both the WT and VCP TG mice at this age.

VCP TG mice and their litter-matched FVB WT male and female mice at three to five months old were randomly assigned into two experimental groups, sham or TAC for 2 weeks. TAC was performed as previously described^10, 36^. The sham-operated mice underwent the same procedure except for constriction of the aorta. Cardiac hypertrophy and function were confirmed by echography and histology. The heart tissues were collected after ex vivo measurements. All animal procedures were performed under the NIH guidance (Guide for the Care and Use of Laboratory Animals, revised 2011), and the Institutional Animal Care and Use Committee of Loma Linda University approved the protocols.

### RNA extraction and RNA-seq

Total RNA was extracted from left ventricular (LV) tissues using the Qiagen miRNeasy kit. NuGen Ovation Mouse RNA-Seq kit was used to construct RNA-seq libraries with 1% of Ambion’s ERCC mix1 spike-in control. RNA-seq libraries were sequenced on Illumina HiSeq 4000 at the Loma Linda University Center for Genomics, 150 × 2 bp, paired-end. We also assess the RNA-seq data quality using the ERCC spiked-in control^37^.

### Bioinformatic analysis of RNA-seq data

- *RNA sequencing and quality control (QC)* The next-generation sequencing (NGS) was used to determine the transcriptomic gene expression alteration modulated by VCP using RNA-seq. The raw fastq data were assessed by FastQC (v0.11.4) and Bioconductor package ShortRead for quality control. The trimming process was performed by trimmomatic v0.35^38^ with the following options: LEADING:20 TRAILING:20 CROP:150 HEADCROP:4 SLIDING WINDOW:4:15 MINLEN:100. After trimming, we performed gene-level and transcript-level analyses, respectively. The mouse GRCm38 and Ensembl Mmusculus. v79 were used as a reference genome and transcript annotation files for the following analysis.
  In the gene-level analysis, the trimmed fastq data were aligned to the reference genome and quantified by Kallisto v0.43.1 with default parameters^39^. In Kallisto, isoform expression for each gene was summed to derive the counts and transcript per million (TPM) values by Bioconductor package tximport. The analysis of differentially expressed genes (DEG) was performed with DESeq2^40^. Genes < 10 counts were discarded for the DEG analysis. The DEGs were defined as the false discovery rate (FDR) < 0.05 or FC > 2 with *p* < 0.05.
  In the transcript-level analysis, the trimmed fastq data were aligned by the 2-pass mode of STAR v2.5.4b^41^ with default parameters. The analysis of DETXs was performed by LeafCutter^11^ with a default parameter setting. The splicing variants were normalized by proportion for differential analysis. Briefly, the leafcutter was applied to the spliced reads to quantify differential intron usage across samples. For details, it first extracted the junction reads from bam files to identify the alternatively excised introns and summarized the intron usage as proportions across samples of two groups. Finally, a differential analysis of intron usage proportions between the two groups' samples was performed using a Dirichlet-multinomial model. A splicing event was labeled significantly if the FDR < 0.05 and LeafViz was used to visualize the significant splicing events.
- *Visualization of differentially expressed genes* To visualize the DEG, we performed a Principal Component Analysis (PCA) and a Hierarchical Clustering Analysis (HCA) by Partek Genomic Suite 6.0 with default options. Circos v 0.69-32^42^ was used to draw the fold change of DEGs under different comparisons.
- *Gene Ontology (GO) functional analysis* GO analysis was performed by using a web-based tool, GeneCodis^43–45^. Briefly, a statistical test, usually the hypergeometric, χ^2^, binomial, or Fisher's exact test, is used to compute *p* values, which are subsequently adjusted for multiple testing. The result of this analysis is a list of single biological annotations from a given ontology with their corresponding *p* values. Those terms with *p* values indicating statistical significance are representative of the analyzed list of genes and can provide information about the underlying biologic processes. Graphs were generated by GraphPad Prism8 based on the GeneCodis data.
- *Pathway analysis* The assessment of biological and interaction networks of candidate genes at 2W TAC within WT and VCP TG were generated through the use of IPA (QIAGEN Inc., https://www.qiagenbioinformatics.com/products/ingenuity-pathway-analysis). The candidate genes were uploaded into the IPA for the identification of their biological function and the functional networks of the eligible molecules.

### Real-time quantitative RT-PCR (q-PCR)

qPCR was used to validate some selective DEGs. cDNA was synthesized from RNA of each sample using the Transcriptor First Strand cDNA Synthesis Kit (Roche). qRT-PCR was performed on a CFX96 Touch Real-Time PCR Detection System by using iTaq Universal SYBR Green Supermix (Bio-Rad) according to the manufacturer’s instructions^8, 10, 21^. Each sample was performed in triplicate and the average value was taken. Gapdh was used as the control to normalize the targeted genes. The sequences of the primers used for qPCR were presented in Table 3.

**Table 3:** The sequences of the primers used for the qPCR for this study.

Genes	Forward primer sequence (5′– > 3′)	Reverse primer sequence (5′– > 3′)
*Olfr1079*	TAGTGACTGAATTTATCCTCAGGG	CATAGGTGTTTGCAGCCTTG
*Olfr1373*	GGAGGGAAAATTTCTTGCCCTTT	GGAGTCTGTGCCTCTCCCTA
*Olfr181*	ACATGAAGCAATCCTGATCTGA	TGTTCAGTTGAGAGGCACATGA
*Olfr787*	CTGGGCATATCAGACGATCCA	GTCCAGCTTGAGGACCAACAT
*Olfr193*	CAGCCAGTGAGGACATGGAAAT	TTGCCCACAATGGTGATGAGA
*Olfr1311*	AAAGCCAATCACTCTGTTGTGT	ACTCGCTATGTAGAACATGGAGG
*Olfr1299*	TTGGGACTTTCCCGCTCAC	GGAGATGGCGGTCAATGGT
*Olfr998*	TGTGGGAACTGAGTGTTTCCT	GCACACATACCCTCTGAGACATA
*Olfr1231*	GGGTCTCACACAGAATCCACG	GTACATTGGAGCTGAAAGGGTG
*Ndufv3-tv1*	GAGAGGGGCAAGCTCCTAAC	ACGCTACCAAAGTCTTTCTTGAC
*Ndufv3-tv2*	CGGGAGAACTGGTTTCTGTAGT	CTCGGGCTCTTTGAGCACA
*Ndufs6-nm*	CAGTTCAAGCAGCACCATCAC	CCAGCGTGGAGATGTTCCATA
*Ndufs6-nr*	GGGGAAAAGATCACGCATACC	TGCTACCGTCAGTCTTGGG
GAPDH	CATGGCCTTCCGTGTTCCTA	CCTGCTTCACCACCTTCTTGAT

### Statistical analysis

Differences among groups were determined by one-way ANOVA followed by a posthoc Tukey test. A value of *p* < 0.05 was considered significant.

## Supplementary information

Supplementary Information

## Data Availability

Our RNA-seq fastq files were submitted to GEO with the access number (GSE134085). The dataset will be available as soon as our manuscript is accepted for publication.
