# Supplementary material for: Novel genomic targets of valosin-containing protein in protecting pathological cardiac hypertrophy
Source: Sci Rep. 2020 Oct 22;10:18098. doi: 10.1038/s41598-020-75128-z (PMC7582185; doi:10.1038/s41598-020-75128-z)
Supplement: Supplementary file 1 — Supplementary Information [file 41598_2020_75128_MOESM1_ESM.pdf]

Supplemental Data (2 Figures; 3 Tables)

Novel genomic targets of valosin-containing protein in protecting pathological cardiac hypertrophy

Ning Zhou[a'b]*, Xin Chen[c]*, Jing Xi[a]*, Ben Ma[a'e], Christiana Leimena[a], Shaunrick Stoll[a], Gangjian Qin[d], Charles Wang[c#], Hongyu Qiu[a'e #]

a. Division of Physiology, Department of Basic Sciences, School of Medicine, Loma Linda University, Loma Linda, CA, 92350 USA
b. Division of Cardiology, Department of Internal Medicine, Tongji Hospital, Tongji Medical College, Huazhong University of Science and Technology, Wuhan, 430000, China
c. Center for Genomics & Department of Basic Sciences, School of Medicine, Loma Linda University, Loma Linda, CA 92350, USA;
d. Department of Biomedical Engineering, School of Medicine and School of Engineering, University of Alabama at Birmingham, Birmingham, AL 35294, USA
e. Center of Molecular and Translational Medicine, Institution of Biomedical Science, Georgia State University, Atlanta, GA 30303, USA

*** Equal contributions**

# Correspondences to:

Hongyu Qiu, MD, PhD
Center of Molecular and Translational Medicine
Institution of Biomedical Science
Georgia State University
Petit Research Center, Room 588
100 Piedmont Ave
Atlanta, GA 30303
Tel: 404 413 3371
Fax: 404-413-9566
Email: hqiu@gsu.edu

Charles Wang, MD, PhD
Center for Genomics
Department of Basic Sciences
School of Medicine
Loma Linda University
11021 Campus Street, AH 120/104
Loma Linda, CA 92350
Phone: 909-558-8759
Fax: 909-558-0464
Email:chwang@llu.edu

Short title: Gene regulation of VCP against cardiac pressure overload

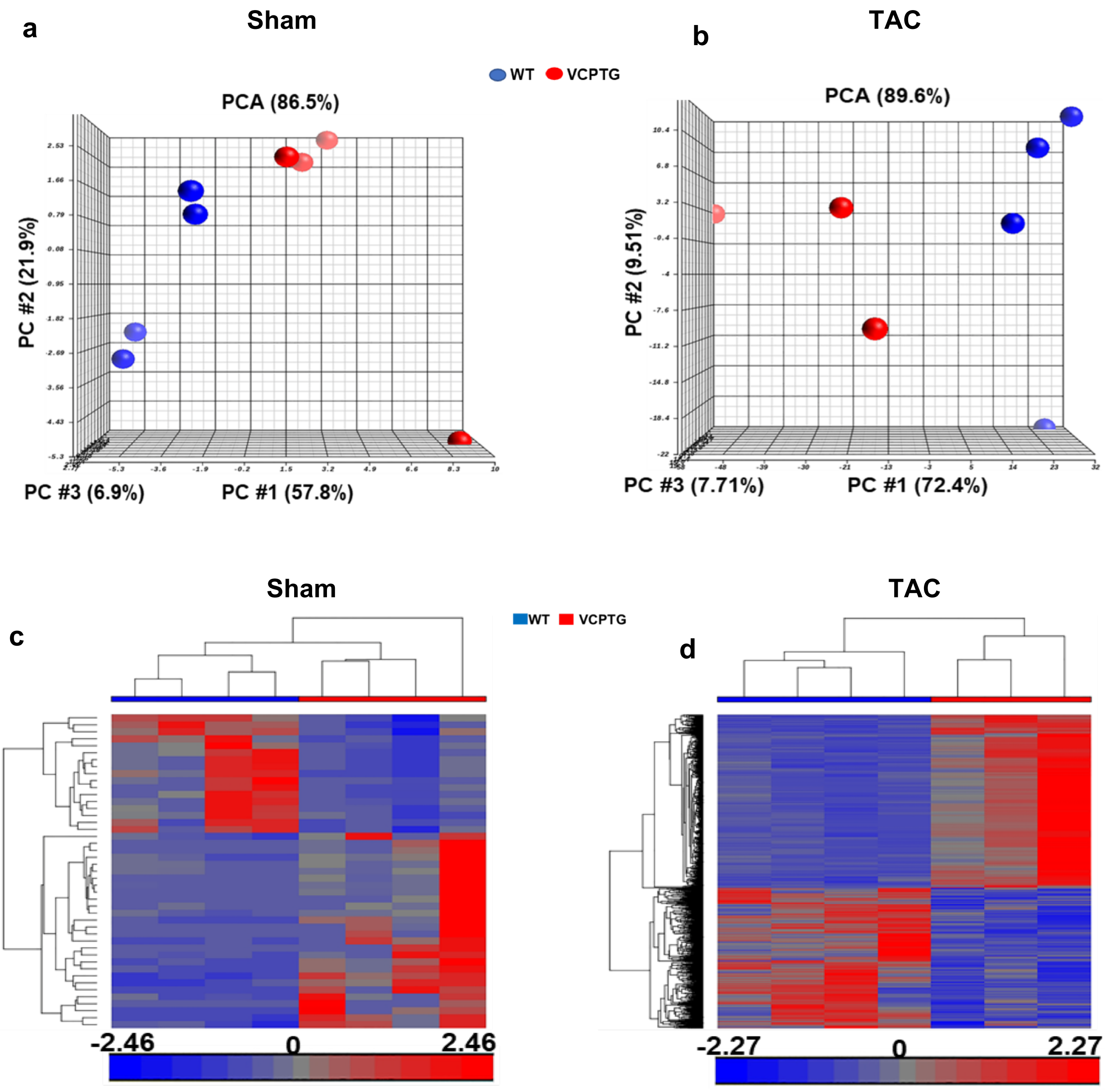

Figure S1. The profiles of the transcriptomic alterations between VCP TG and WT mice at the sham and 2 weeks TAC conditions. a-b. Principal component analysis (PCA) and **c-d.** Hierarchical clustering of differentially expressed genes (DEGs) between VCP TG and WT mice with FDR< 0.05 in sham (a and c) and 2W TAC (b and d). N=3-4/group.

Table S1. Top DEGs between VCP TG and WT by fold changes

sham					2 weeks TAC				
Transcript ID	log2 FC	P value	FDR	Genes	Transcript ID	log2 FC	P value	FDR	Genes
ENSMUSG00000030046	10.07	5.07E-07	2.312E-03	*Bmp10*	ENSMUSG00000099620	5.88	2.23E-04	9.00E-03	*Mrgprb13*
ENSMUSG00000083327	8.68	3.40E-08	4.620E-04	*Vcp-rs*	ENSMUSG00000102781	5.07	3.13E-03	4.40E-02	*Pla2r1*
ENSMUSG00000041616	5.11	3.90E-05	2.830E-02	*Nppa*	ENSMUSG00000009356	4.91	1.74E-04	7.00E-03	*Lpo*
ENSMUSG00000049436	4.32	3.31E-05	2.805E-02	*Upk1b*	ENSMUSG00000027376	4.81	8.93E-06	1.00E-03	*Prom2*
ENSMUSG00000069476	4.26	3.12E-05	2.805E-02	*Zfp616*	ENSMUSG00000020805	4.45	3.16E-04	1.10E-02	*Slc13a5*
ENSMUSG00000039419	3.90	4.50E-05	3.127E-02	*Cntnap2*	ENSMUSG00000042002	4.33	3.96E-04	1.20E-02	*Foxn4*
ENSMUSG00000027867	3.89	3.46E-05	2.805E-02	*Spag17*	ENSMUSG00000050201	4.07	2.81E-03	4.10E-02	*Otop2*
ENSMUSG00000039963	3.84	6.04E-07	2.312E-03	*Ccdc40*	ENSMUSG00000020159	3.64	4.51E-05	3.00E-03	*Gabrp*
ENSMUSG00000073940	3.67	9.86E-05	4.569E-02	*Hbb-bt*	ENSMUSG00000021441	3.61	1.22E-04	6.00E-03	*Cts6*
ENSMUSG00000025889	3.63	4.75E-08	4.620E-04	*Snca*	ENSMUSG00000017950	3.42	1.89E-03	3.20E-02	*Hnf4a*
ENSMUSG00000079014	-3.30	1.68E-05	1.814E-02	*Serpina3i*	ENSMUSG00000061295	3.40	1.65E-03	3.00E-02	*Olfr1261*
ENSMUSG00000043410	3.21	9.63E-05	4.568E-02	*Hfm1*	ENSMUSG00000073952	3.40	1.18E-03	2.40E-02	*Olfr597*
ENSMUSG00000006403	-3.05	5.35E-05	3.473E-02	*Adamts4*	ENSMUSG00000025576	3.38	1.65E-03	3.00E-02	*Rbfox3*
ENSMUSG00000030616	3.03	4.02E-06	7.120E-03	*Sytl2*	ENSMUSG00000000103	3.19	1.55E-03	2.80E-02	*Zfy2*
ENSMUSG00000075249	2.87	7.82E-06	1.015E-02	*Fsip2*	ENSMUSG00000061457	3.19	2.29E-04	9.00E-03	*Olfr871*
ENSMUSG00000025473	-2.79	4.56E-06	7.389E-03	*Adam8*	ENSMUSG00000034552	3.19	2.41E-05	2.00E-03	*Zswim2*
ENSMUSG00000034533	2.60	1.07E-06	2.597E-03	*Scn10a*	ENSMUSG00000040728	3.06	1.09E-04	5.00E-03	*Esrp1*
ENSMUSG00000062991	2.34	5.62E-05	3.527E-02	*Nrg1*	ENSMUSG00000022416	2.98	1.05E-03	2.30E-02	*Cacna1i*
ENSMUSG00000025902	-2.11	8.41E-06	1.023E-02	*Sox17*	ENSMUSG00000027457	2.98	7.27E-04	1.80E-02	*Snph*
ENSMUSG00000030748	-2.06	1.06E-05	1.216E-02	*Il4ra*	ENSMUSG00000096284	2.95	1.27E-03	2.50E-02	*Tcstv1*

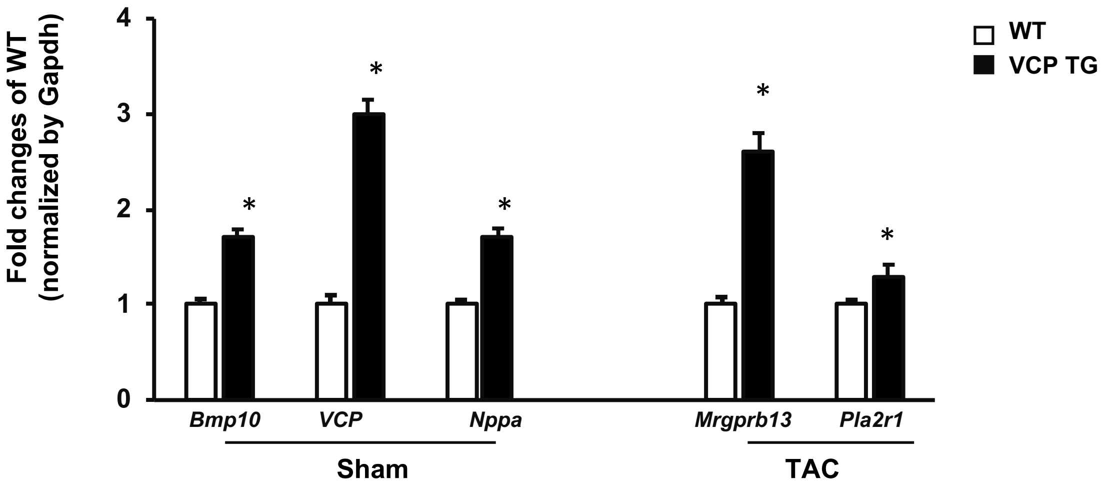

Figure S2. The validation of the alterations of the top DEGs between VCP TG vs WT by qPCR. *, $p < 0.05$ vs WT. N=4-5/groups

Table S2. DEGs detected between VCP TG vs WT at sham condition based on FDR<0.05 and their corresponding alterations under 2W TAC based on fold changes

Gene symbol	*Sham: VCP TG vs WT log2FoldChange*	*2W TAC: VCP TG vs WT log2FoldChange*	*WT: 2W TAC vs sham log2FoldChange*
Hlf	1.285	0.515	-0.461
Adamts4	-3.047	0.015	2.162
Rgcc	-1.864	-0.433	1.739
Angpt1	1.706	0.2	-1.103
Tfrc	1.083	0.26	-0.537
Fas	-1.835	-0.5	1.2
Adam8	-2.789	1.049	3.026
Snca	3.634	-0.516	-1.086
Sox17	-2.108	-0.042	2.336
Epha4	1.515	0.537	-0.482
Kcnj3	1.684	-0.108	-1.398
Mamdc4	**-1.65**	**1.066**	**1.577**
Spag17	3.887	0.373	-4.449
Vcp	1.051	0.78	-0.278
Tinagl1	-1.621	0.099	1.537
Lrrc47	-1.036	0.1	1.305
Bmp10	10.073	0.663	NA
Sytl2	3.031	0.454	-2.885
Il4ra	-2.064	-0.443	1.724
Tacc2	-1.417	0.252	1.383
Plscr2	-1.334	-0.591	1.213
Scn10a	2.602	0.892	NA
Aldh1b1	1.976	0.35	-1.104
Sh3tc1	-1.463	0.131	1.665
Prdm16	-0.941	-0.699	0.954
Cntnap2	**3.904**	**2.009**	**-2.954**
Ccdc40	3.843	-1.444	-3.652
Spns2	-1.425	0.139	1.474
Fam117b	**-1.548**	**-0.866**	**0.729**
Nppa	5.106	-1.122	NA
Hfm1	**3.215**	**1.731**	**-2.543**
BC049715	**3.858**	**1.93**	**-2.716**
Upk1b	4.315	-0.573	-6.224
2810025M15Rik	-1.382	0.202	0.619
Erbb4	1.614	-0.074	-1.216
Nrg1	2.342	-2.083	NA
Zfp616	**4.265**	**1.831**	**-3.345**
Hbb-bt	3.666	-0.084	-1.355
Dcdc5	1.746	0.04	-2.559
Fsip2	**2.874**	**1.969**	**-2.937**
Serpina3i	-3.295	-0.674	1.847
Gm7901	6.887	2.252	-4.217
Vcp-rs	8.68	2.728	NA
Gm3099	6.176	5.232	NA
Gm16513	4.469	1.65	-3.986

Table S3. The DETX between VCP TG and WT in different comparisons

Sham: VCP TG vs WT				2W TAC: VCP TG vs WT				WT: 2W TAC vs Sham			
ETX_cluster	DETX_gen	DETX	DETX_p.adj	DETX_cluster	DETX_ge	DETX	DETX_p.adj	DETX_cluster	DETX_ge	DETX	DETX_p.adju
chr4:clu_504_NA	*Asph*	10	0.00093	chr2:clu_3453_NA	***Ttn***	29	8.80E-10	chr4:clu_483_NA	*Asph*	10	2.0955E-06
chr7:clu_1121_NA	*Tacc2*	13	0.01753	chr11:clu_4241_NA	*Obscn*	23	0.00565	chr19:clu_2990_NA	***Sorbs1***	9	2.7497E-05
chr19:clu_2969_NA	***Sorbs1***	11	0.0169	chr2:clu_3460_NA	***Ttn***	7	8.55E-06	chr7:clu_883_NA	*Fxyd1*	5	1.3969E-05
chr17:clu_444_NA	*Ptprm*	5	0.01056	chr16:clu_2899_NA	*Eif4g1*	7	0.00344	chr8:clu_2265_NA	*Sorbs2*	8	0.00019223
chr4:clu_678_NA	*Epb4.1*	4	0.02129	chr7:clu_753_NA	*Fxyd1*	8	0.00565	chr16:clu_3533_NA	*Srl*	4	2.482E-05
chr15:clu_1439_NA	*Pacsin2*	4	0.02588	chr19:clu_2429_NA	***Sorbs1***	9	0.00913	chr3:clu_5013_NA	*Myoz2*	7	0.00050803
chr10:clu_3309_NA	*Sar1a*	2	0.0169	chr6:clu_3840_NA	*Wnk1*	7	0.00605	chr5:clu_2071_NA	*Myl2*	5	0.00037213
chr7:clu_1019_NA	*Serpinh1*	2	0.0169	chr1:clu_2117_NA	*Tns1*	5	0.00312	chr10:clu_3454_NA	*Mrpl42*	2	2.482E-05
chr17:clu_440_NA	*Ndufv2*	2	0.0169	chr16:clu_2939_NA	*Golgb1*	6	0.00673	chr5:clu_1878_NA	*Hadhb*	6	0.00137584
chr7:clu_1165_NA	*Nap1l4*	2	0.0169	chr19:clu_2423_NA	***Sorbs1***	6	0.02871	chr5:clu_1990_NA	*Sec31a*	6	0.00534863
chr8:clu_2450_NA	*Sipa1l2*	2	0.02356	chr9:clu_3257_NA	*Clasp2*	2	0.00312	chr17:clu_385_NA	*Apobec2*	3	0.00137584
chr16:clu_3560_NA	*Comt*	2	0.02588	chr11:clu_4296_NA	*Eno3*	5	0.0312	chr11:clu_5105_NA	*Ogdh*	2	0.00050803
chr18:clu_1748_NA	*Arap3*	2	0.02588	chr1:clu_2180_NA	*Ppip5k2*	5	0.03347	chr19:clu_2932_NA	*Prune2*	3	0.00156037
chr2:clu_4214_NA	***Ttn***	2	0.03074	chr8:clu_1774_NA	*Insr*	2	0.00497	chrX:clu_1175_NA	*Kdm6a*	4	0.00398873
chr16:clu_3590_NA	*Dlg1*	2	0.04115	chr10:clu_2689_NA	*Pln*	5	0.04452	chr1:clu_2687_NA	*Ppip5k2*	5	0.0129382
chr9:clu_3732_NA	*Dnm2*	2	0.04634	chr9:clu_3277_NA	*Trak1*	2	0.00789	chr10:clu_3439_NA	*Chpt1*	5	0.01333318
chr1:clu_2653_NA	*Hdac4*	1	0.04115	chr10:clu_2784_NA	*Chpt1*	4	0.03976	chr8:clu_2453_NA	*Zc3h18*	2	0.0011584
chr14:clu_176_NA	*Kctd9*	1	0.04479	chr7:clu_808_NA	*Pex11a*	4	0.04073	chr1:clu_2611_NA	*Tns1*	6	0.0237929
				chr14:clu_118_NA	*Hnrnpc*	3	0.02598	chr10:clu_3503_NA	*Naca*	2	0.00156037
				chr17:clu_266_NA	***Ndufv3***	2	0.01153	chr16:clu_3630_NA	*Golgb1*	6	0.03618515
				chr14:clu_132_NA	*Myh6*	3	0.0312	chr14:clu_124_NA	*Hnrnpc*	3	0.00535741
				chr10:clu_2781_NA	*Igf1*	2	0.01354	chr7:clu_802_NA	*Rdh13*	2	0.0026096
				chr18:clu_1458_NA	*Csnk1a*	2	0.0229	chr6:clu_4548_NA	*Ndufa5*	2	0.00269151
				chr4:clu_612_NA	*Hp1bp3*	3	0.04073	chr3:clu_5057_NA	*Nexn*	4	0.01661022
				chr14:clu_52_NA	*Ppif*	2	0.0256	chrX:clu_1212_NA	*Idh3g*	4	0.01661022
				chr1:clu_2166_NA	*Lrrfip1*	2	0.03951	chr2:clu_4202_NA	*March7*	2	0.003058
				chr5:clu_1565_NA	*Ppargc1*	2	0.03976	chr1:clu_2652_NA	*Sp100*	5	0.03618515
				chr17:clu_235_NA	*Hagh*	2	0.04073	chr17:clu_328_NA	*Brd2*	3	0.01228053
				chr7:clu_937_NA	*Tollip*	2	0.04361	chr16:clu_3601_NA	*Opa1*	2	0.00535741
				chr8:clu_1956_NA	*Chtf8*	2	0.04452	chr17:clu_360_NA	*Rcan2*	2	0.00535741
				chr5:clu_1585_NA	*Ociad1*	2	0.04452	chr17:clu_351_NA	*Rpp21*	2	0.00535741
				chr6:clu_3794_NA	*Iqsec1*	1	0.02477	chr11:clu_5302_NA	*Myo18a*	2	0.00811435
				chr4:clu_445_NA	*Npr2*	1	0.0256	chr1:clu_2803_NA	*Dcaf6*	2	0.00986572
				chr3:clu_3984_NA	*Mrpl24*	1	0.02598	chr7:clu_929_NA	*Ube3a*	2	0.01117853
				chr13:clu_1310_NA	***Ndufs6***	1	0.02851	chr12:clu_3050_NA	*Ncoa1*	4	0.04211347
				chr5:clu_1629_NA	*Nudt9*	1	0.03976	chr9:clu_3965_NA	*Rbm5*	2	0.01228053
				chr7:clu_916_NA	*Fam53b*	1	0.04073	chr18:clu_1752_NA	*Arap3*	2	0.01661022
				chr5:clu_1668_NA	*Tesc*	1	0.04073	chrX:clu_1233_NA	*Apoo*	2	0.01661022
				chr7:clu_862_NA	*Ipo7*	1	0.04073	chr2:clu_4203_NA	*Itgb6*	2	0.01661022
								chr14:clu_52_NA	*Ppif*	2	0.01661022
								chr10:clu_3331_NA	*Sar1a*	2	0.0176823
								chr16:clu_3590_NA	*Tra2b*	2	0.02135337
								chr8:clu_2335_NA	*Arhgap10*	2	0.02136414
								chr7:clu_997_NA	*Serpinh1*	3	0.04274286
								chr13:clu_1513_NA	*Mtr*	2	0.0237929
								chr16:clu_3657_NA	*Cblb*	3	0.04785809
								chr2:clu_4260_NA	*Ccdc141*	2	0.02739385
								chr5:clu_2007_NA	*Gbp6*	2	0.03065032
								chr2:clu_4165_NA	*Dab2ip*	2	0.03065032
								chr1:clu_2601_NA	*Acadl*	2	0.03068008
								chr2:clu_4250_NA	***Ttn***	2	0.03068008
								chr2:clu_4450_NA	*Rbm39*	2	0.03618515
								chr4:clu_575_NA	*Nfia*	2	0.03618515
								chrX:clu_1301_NA	*Msl3*	2	0.03826999
								chr18:clu_1807_NA	*Tcf4*	2	0.03826999
								chr7:clu_1145_NA	*Nap1l4*	2	0.03871731
								chr5:clu_1929_NA	*Pcdh7*	1	0.01654877
								chr18:clu_1778_NA	*Synpo*	2	0.0400688
								chr8:clu_2366_NA	*Dnase2a*	1	0.01661022
								chr15:clu_1354_NA	*Tmem65*	2	0.04211347
								chr7:clu_1083_NA	*Tgfb1i1*	2	0.04211347
								chr6:clu_4690_NA	*Setd5*	2	0.04274286
								chr7:clu_1052_NA	*Ndufab1*	2	0.04274286
								chr17:clu_318_NA	*Hnrnpm*	2	0.04274286
								chr12:clu_3149_NA	*Srsf5*	2	0.04274286
								chr18:clu_1781_NA	*Camk2a*	2	0.04274286
								chr1:clu_2686_NA	*Ppip5k2*	1	0.01741987
								chr19:clu_2970_NA	*Btaf1*	1	0.0176823
								chr3:clu_4935_NA	*Ubap2l*	2	0.04971823
